# FOXO1 re-expression with a dual-recombinase allele rescues class switching in germinal center B cells

**DOI:** 10.1084/jem.20241136

**Published:** 2025-12-26

**Authors:** Carlota Farré Díaz, Eleni Kabrani, Wiebke Winkler, Eric Blanc, Brigitte Wollert-Wulf, Claudia Salomon, F. Thomas Wunderlich, Dieter Beule, Martin Janz, Klaus Rajewsky

**Affiliations:** 1 Immune Regulation and Cancer, Max Delbrück Center for Molecular Medicine in the Helmholtz Association, Berlin, Germany; 2 Biology of Malignant Lymphomas, Max Delbrück Center for Molecular Medicine in the Helmholtz Association, Berlin, Germany; 3 Experimental and Clinical Research Center, a cooperation between the Max Delbrück Center for Molecular Medicine in the Helmholtz Association and the Charité – Universitätsmedizin Berlin, Berlin, Germany; 4 Hematology, Oncology and Cancer Immunology, Charité – Universitätsmedizin Berlin, Berlin, Germany; 5 Genome Diversification & Integrity, Max Delbrück Center for Molecular Medicine in the Helmholtz Association, Berlin, Germany; 6 Core Unit Bioinformatics, Charité – Universitätsmedizin Berlin, corporate member of Freie Universität Berlin and Humboldt-Universität zu Berlin and Berlin Institute of Health, Berlin, Germany; 7 Faculty of Medicine and University Hospital Cologne, Center for Molecular Medicine Cologne, University of Cologne, Cologne, Germany; 8 Cologne Excellence Cluster for Stress Responses in Ageing-Associated Diseases, Center for Molecular Medicine, University of Cologne, Cologne, Germany; 9 Max-Planck-Institute for Metabolism Research, Cologne, Germany

## Abstract

Modeling complex (patho)physiological processes by sequential targeted mutagenesis in mice is limited by the lack of precision of cellular targeting and complex breeding strategies. We present a new Cre/DreERT2 dual-recombinase germinal center B cell (GCBC)–specific strain, with co-expression of the recombinases from a single allele. This enables highly efficient Cre-mediated FOXO1 knockout in GCBCs *in vivo*, followed by time-controlled, efficient Dre-mediated FOXO1 re-expression in the same cells, leading to functional rescue of GC compartmentalization and class switch recombination. The present approach can be easily adapted to other cellular contexts.

## Teaser

Farré Díaz et al. describe a broadly applicable system for sequential targeted mutagenesis using two recombinases sequentially activated from a single locus. As a proof-of-concept experiment, the time-controlled re-expression of FOXO1 rescued the defects associated with FOXO1-deficient germinal center B cells, including class switch recombination, providing new insights into FOXO1 biology via a dual-recombinase tool.

## Graphical Abstract

**Figure:**
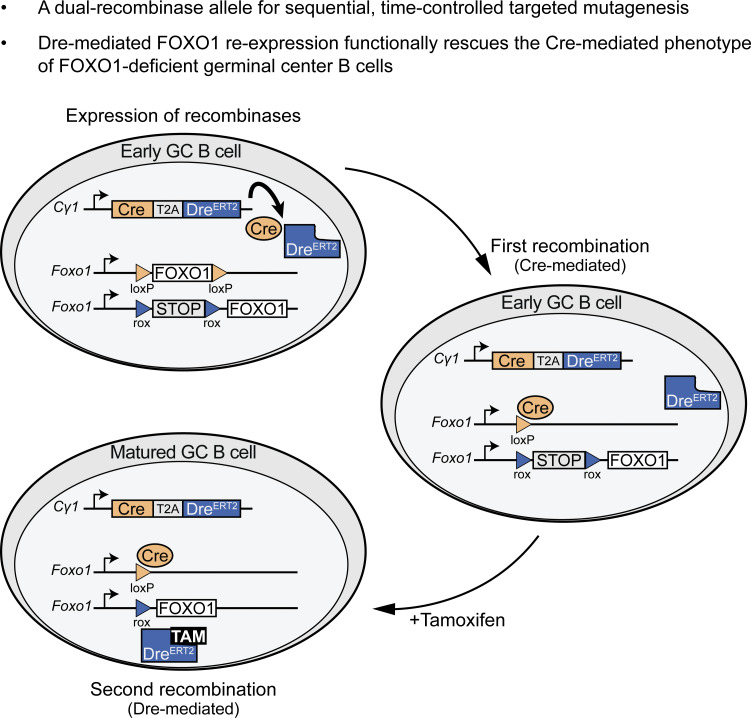

## Introduction

Site-specific recombination systems, such as Cre/loxP, are key technologies to study gene function and (patho)physiological processes *in vivo*. Dual-recombinase approaches represent a further technical advance that enables more refined disease modeling, incorporating multistep genetic manipulations and lineage tracing (Li et al., 2023; Sket et al., 2023). However, one limitation of the currently available systems is that the recombinases used are expressed from different loci, thereby requiring the generation of complex compound mutant mice—containing separate transgenes encoding the recombinases together with the respective target alleles—and thus complicating the analysis in terms of both breeding time frame and fidelity of sequential mutagenesis at the level of individual cells. Here, we describe a dual-recombinase mouse strain in the context of germinal center (GC) B cells (GCBCs), where Cre and the tamoxifen (TAM)-dependent DreERT2 recombinases are co-expressed from the immunoglobulin heavy constant gamma 1 (*Ighg1/Cγ1*) locus and thus at the initiation of the GC response.

Upon antigen encounter, mature B cells undergo clonal expansion in distinct histological structures called GCs. In the course of the GC reaction, GCBCs diversify their immunoglobulin repertoire via class switch recombination (CSR) and somatic hypermutation (SHM) of antibody V region genes (Victora and Nussenzweig, 2022). Positively selected GCBCs differentiate into memory B cells (MBCs) or antibody-secreting plasma cells (PCs), thus shaping the humoral immune response (Victora and Nussenzweig, 2022). Both CSR and SHM require the introduction and efficient repair of DNA strand breaks in the immunoglobulin loci (Chi et al., 2020). Errors during these processes can lead to oncogenic mutations and/or translocations, rendering (post)-GCBCs the origin of most B cell malignancies (Küppers et al., 1999). In order to study the physiology and malignant transformation of GCBCs, a number of Cre mouse lines have been generated (Casola et al., 2006; Crouch et al., 2007; Dogan et al., 2009; Shinnakasu et al., 2016; Weber et al., 2019; Callahan et al., 2024). However, none of the currently available lines allows for the genetic manipulation of GCBC differentiation in a stepwise manner. This technical limitation prevents not only the *in vivo* investigation of the role and cooperation of genes in different phases of the GC reaction, but also the study of the sequential acquisition of oncogenic events in this microenvironment, underlying malignant transformation. In this study, by sequentially targeting the endogenous *Foxo1* locus during the GCBC reaction as a proof-of-concept experiment using a novel dual-recombinase mouse strain, we demonstrate highly efficient Cre-mediated FOXO1 knockout (KO) with perturbation of GC physiology, followed by time-controlled Dre-mediated FOXO1 re-expression and phenotypic rescue, demonstrating that this system can be used *in vivo* for sequential targeted mutagenesis that is precisely controlled with respect to cellular compartment and time.

## Results

### Efficient Cre-mediated followed by Dre-mediated recombination using the GCBC-specific *Cγ1-Cre_T2A_DreERT2* (*Cγ1-CDE*) strain

**Figure 1.**
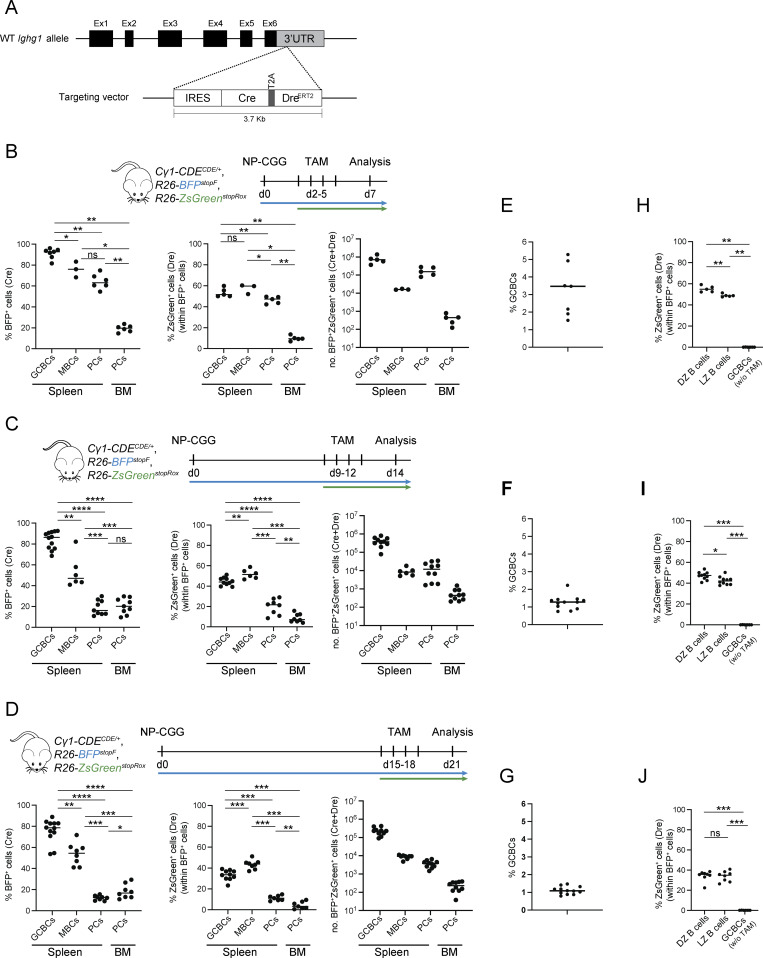
**Characterization of the *Cγ1-CDE* strain. (A)** Targeting strategy for the insertion of the IRES-Cre_T2A_DreERT2 cassette into the 3′-UTR downstream of the last membrane exon of the mouse *Cγ1* locus. **(B–J)** Experimental scheme using the *Cγ1-CDE, R26-BFP*^*stopF*^*, R26-ZsGreen*^*stopRox*^ compound mutant mice, immunizing with NP-CGG at day (d)0, administrating TAM at day 2–5 (B), 9–12 (C), or 15–18 (D) and analyzing at day 7, 14, or 21, respectively. **(B–D)** Percentage of Cre-mediated BFP^+^ (left panel) and Dre-recombined ZsGreen^+^ (within the BFP^+^) cells (middle panel) among splenic Fas^+^CD38^−^ GCBCs, splenic class-switched Fas^−^CD38^+^IgD^−^IgG1^+^ MBCs, and CD138^+^TACI^+^ PCs from spleen and bone marrow (BM) (from one femur and tibia), as well as corresponding absolute numbers of the BFP^+^ZsGreen^+^ cells (right panel). **(E–G)** Percentage of Fas^+^CD38^−^ GCBCs at day 7 (E), day 14 (F), and day 21 (G) after NP-CGG immunization. **(H–J)** Percentage of ZsGreen^+^ (within BFP^+^) CXCR4^hi^DC86^low^ DZ and CXCR4^low^CD86^hi^ LZ GCBCs at day 7 (H), day 14 (I), and day 21 (J) after NP-CGG immunization. Data points for GCBCs without TAM administration are compiled from the three time points analyzed. Statistics: Mann–Whitney test, *P ≤ 0.05; **P ≤ 0.01; ***P ≤ 0.001; ****P ≤ 0.0001; ns, not significant. Each dot represents one mouse. Data are from at least two independent experiments. Horizontal lines indicate the median.

**Figure S1.**
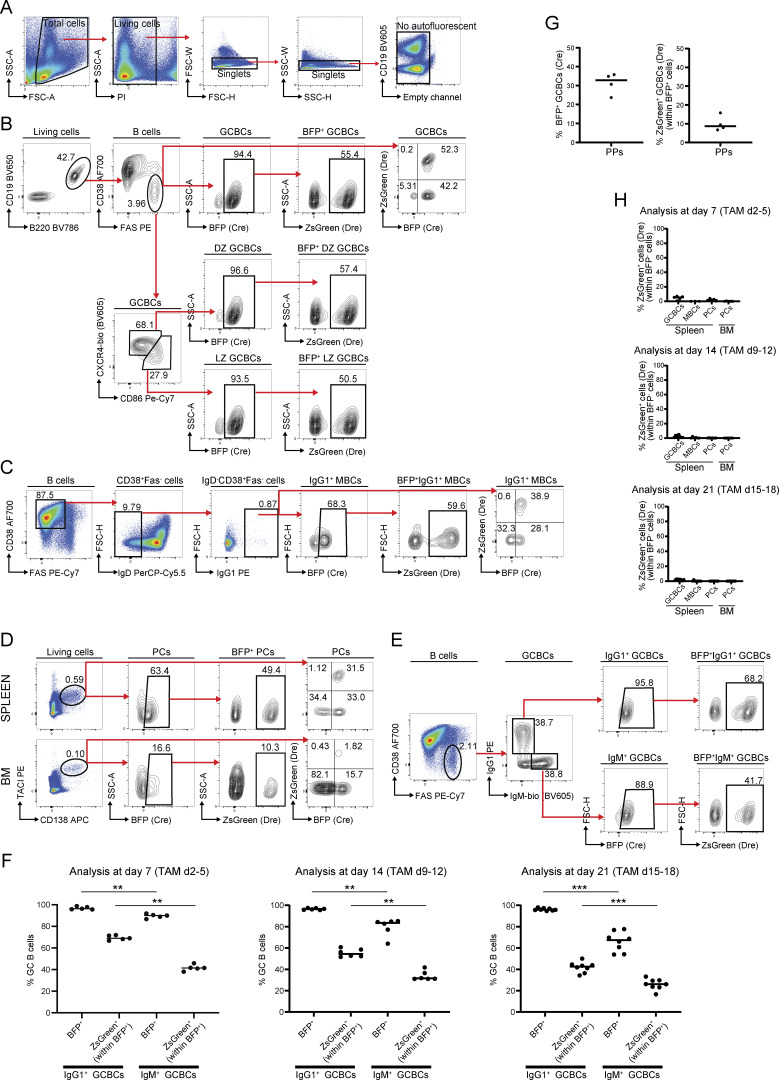
**Characterization of the *Cγ1-CDE* strain. (A–E)** Representative gating strategies for non-autofluorescence living single cells (A) and reporter-positive GCBCs (B)—including DZ and LZ GCBCs; IgG1^+^ MBCs (C); PCs (D); and IgG1^+^/IgM^+^ GCBCs (E). **(F)** Quantification of E at day 7—TAM day 2–5 (left panel); day 14—TAM day 9–12 (middle panel); and day 21—TAM day 15–18 (right panel) after NP-CGG immunization. **(G)** Percentage of Cre-mediated BFP^+^ (left panel) and Dre-mediated ZsGreen^+^ (within the BFP^+^) cells (right panel) among Fas^+^CD38^−^ GCBCs in PPs. **(H)** Percentage of Dre-mediated ZsGreen^+^ (within the BFP^−^) cells among splenic Fas^+^CD38^−^ GCBCs, splenic Fas^−^CD38^+^IgD^−^IgG1^+^ MBCs, and CD138^+^TACI^+^ PCs from spleen and BM (from one femur and tibia) at day 7—TAM day 2–5 (upper panel); day 14—TAM day 9–12 (middle panel); and day 21—TAM day 15–18 (lower panel). Statistics: Mann–Whitney test, **P ≤ 0.01; ***P ≤ 0.001; ns, not significant. Each dot represents one mouse. Data are from at least two independent experiments. Horizontal lines indicate the median.

We have generated a novel dual-recombinase GCBC-specific mouse strain, in which Cre and the TAM-inducible DreERT2 recombinases are concomitantly expressed from the *Ighg1/Cγ1* locus, hereafter called the *Cγ1-CDE* strain (Fig. 1 A). *Cγ1* encodes the constant region of IgG1, and the transgene cassette is inserted in the 3′ untranslated region (UTR), downstream of the last membrane exon. Upon T cell–dependent immunization, most activated B cells that enter the GC produce germline ɣ1 transcripts in response to IL-4 (Casola et al., 2006), allowing the expression of both recombinases—and thus Cre activity—from the *Cγ1* locus early on. Cells that have completed switch recombination to IgG1 continue to express the recombinases (from the nonproductive allele) during the course of the GC reaction (Casola et al., 2006). Since DreERT2 activation is dependent on TAM administration, the second (Dre-mediated) recombination event can be induced in a time-controlled manner, selectively in cells having undergone Cre induction. To functionally validate the newly generated strain, *Cγ1-CDE* mice were crossed to the fluorescent reporter strains *R26-BFP*^*stopF*^ (Sommermann et al., 2020) and *R26-ZsGreen*^*stopRox*^ (Biglari et al., 2021) for detection of Cre- and Dre-mediated recombination, respectively. *Cγ1-CDE*, *R26-BFP*^*stopF*^*, R26-ZsGreen*^*stopRox*^ compound mutant mice were immunized with 4-hydroxy-3-nitrophenylacetyl-conjugated chicken gamma globulin (NP-CGG), and TAM was administered at days 2–5, 9–12, or 15–18 after immunization with subsequent flow cytometry analysis at days 7, 14, or 21, respectively (Fig. 1, B–J). Consistent with the previous characterization of the *Cγ1-Cre* allele (Casola et al., 2006), up to 96% of splenic GCBCs were labeled through Cre-mediated recombination (BFP^+^ GCBCs, Fig. 1, B–D; and Fig. S1, A and B). Further gating on these BFP^+^ GCBCs showed robust and highly efficient Dre-mediated recombination at all time points analyzed (Fig. 1, B–D), with the highest dual-labeling efficiency on day 7 of analysis (up to 60% ZsGreen^+^ cells within the BFP^+^ GCBC population, Fig. 1 B). Importantly, the number of ZsGreen^+^ cells within the BFP^−^ population was negligible at all time points analyzed (Fig. S1 H), demonstrating that Dre-mediated recombination only occurs in cells which have undergone Cre-mediated recombination. GCs are organized into two phenotypically and functionally distinct compartments, the dark zone (DZ) and the light zone (LZ) (Victora and Nussenzweig, 2022). GCBCs in both compartments were successfully labeled (Fig. 1, H–J and Fig. S1 B). In the absence of TAM, no Dre recombination was detected, demonstrating that Dre-mediated recombination is tightly controlled *in vivo* (Fig. 1, H–J). Of note, GCBCs in Peyer’s patches (PPs) could also be labeled, but at lower levels than splenic GCBCs (around 30% BFP^+^ cells; and 10% ZsGreen^+^ within the BFP^+^ population), similar to the lower Cre-mediated recombination levels previously described in PPs for the original *Cγ1-Cre* allele (Casola et al., 2006) (Fig. S1 G).

Germline ɣ1 transcripts (ɣ1 GLTs) are detected in the activated B cell pool early upon immunization, irrespective of whether the cells eventually switch to IgG1 expression (Casola et al., 2006; Roco et al., 2019) Accordingly, Cre-mediated followed by Dre-mediated sequential mutagenesis was achieved not only in IgG1^+^ (up to 70% ZsGreen^+^ within BFP^+^ cells) but also in IgM^+^ (up to 42% ZsGreen^+^ within BFP^+^ cells) GCBCs in our mouse model at all three time points analyzed (Fig. S1, E and F).

As expected, labeling also extends to post-GC cells. Within the MBC compartment, class-switched IgG1^+^ MBCs exhibited solid Cre- and Dre-mediated sequential mutagenesis (up to 84% BFP^+^ cells and 60% ZsGreen^+^ within BFP^+^ cells, Fig. 1, B–D and Fig. S1 C). Analysis of the splenic plasmablast/PC compartment (Fig. 1, B–D and Fig. S1 D) exhibited the highest labeling efficiency for this compartment at day 7 of analysis (up to 75% BFP^+^ cells and 50% ZsGreen^+^ within BFP^+^ cells, Fig. 1 B), indicating the feasibility to target and thus study extrafollicular PCs with our *Cγ1-CDE* strain. However, the fraction of labeled PCs was reduced when analyzing the immunized mice at later time points (days 14 and 21, Fig. 1, C and D), possibly due to the short lifespan of extrafollicular PCs.

### Re-expression of FOXO1 rescues GC compartmentalization and CSR in FOXO1-KO GCBCs

**Figure S2.**
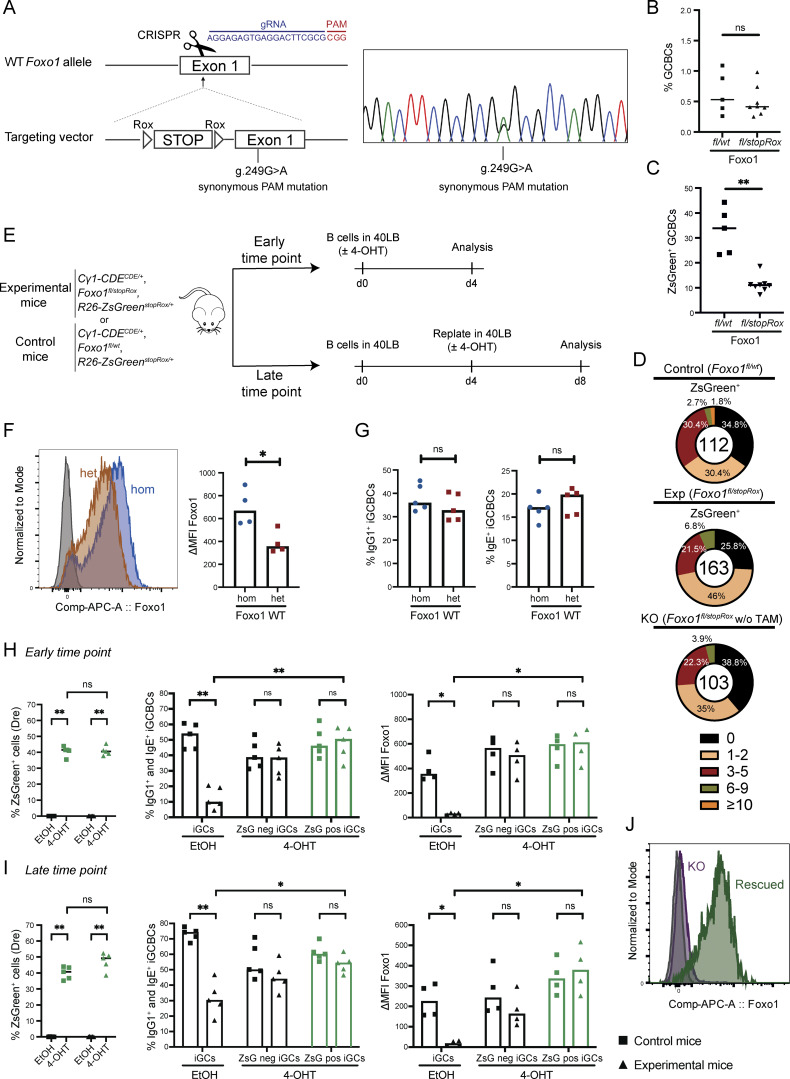
**Sequential mutagenesis at the endogenous *Foxo1* locus. (A)** Targeting strategy at the endogenous *Foxo1* locus. A STOP cassette flanked by rox sites was introduced in front of the endogenous Kozak sequence, and the sequence of exon 1 was modified in order to contain a synonymous PAM mutation in position g.249G>A, which abrogated the genetic editing of the targeting vector used for the homologous recombination. The gRNA sequence used is shown in blue. Heterozygous mutant mice were verified by PCR and Sanger sequencing that allowed for the detection of the PAM mutation and thus verification of successful targeting. **(B and C)** Percentage of total GCBCs (B) and ZsGreen^+^ GCBCs (C) from control *Foxo1*^*fl/wt*^ and experimental *Foxo1*^*fl/stopRox*^ mice at day 14 (TAM day 10–12) after immunization. **(D)** Pie charts showing the mutational load at day 14 (TAM day 10–12) after immunization of *Foxo1*^*fl/wt*^ control, *Foxo1*^*fl/stopRox*^ experimental, and *Foxo1*^*fl/stopRox*^ KO (without TAM administration) ZsGreen^+^ GCBCs (for Foxo1 KO condition, total GCBCs were sorted) from 3 to 5 mice per condition. Number inside the pie chart indicates total number of sequences analyzed. **(E)** Experimental scheme for the analysis of CSR events *in vitro*, inducing Dre recombination (medium supplemented with 4-OHT) at day (d)0 (early time point) or day 4 (late time point) of coculture with 40LB feeder cells (+IL-4), and analyzing at day 4 or day 8, respectively. **(F)** Left panel: representative flow cytometry histogram showing Foxo1 intracellular expression in Foxo1 WT hom (*Foxo1*^*wt/wt*^—blue) and Foxo1 WT het (*Foxo1*^*fl/wt*^—brown) iGCBCs at day 4 of coculture. Foxo1 FMO is depicted in gray. Right panel: quantification of the MFI of Foxo1 subtracting the FMO. **(G)** Percentage of IgG1^+^ (left) or IgE^+^ (right) iGCBCs at day 4 in Foxo1 WT cells. **(H and I)** Percentage of ZsGreen^+^ iGCBCs (iGCs; left panel), percentage of IgG1^+^ and IgE^+^ iGCBCs (middle panel), and Foxo1 MFI in iGCBCs (right panel) at (H) day 4—early time point—or at (I) day 8—late time point—of analysis. **(J)** Representative flow cytometry histogram showing Foxo1 intracellular expression in EtOH-treated (KO) vs. 4-OHT–treated (rescued) iGCBCs from experimental mice. Statistics: Mann–Whitney test, *P ≤ 0.05; **P ≤ 0.01; ns, not significant. Each dot represents one mouse. Data are from at least two independent experiments. Horizontal lines indicate the median. FMO, fluorescence minus one; MFI, median fluorescence intensity.

**Figure 2.**
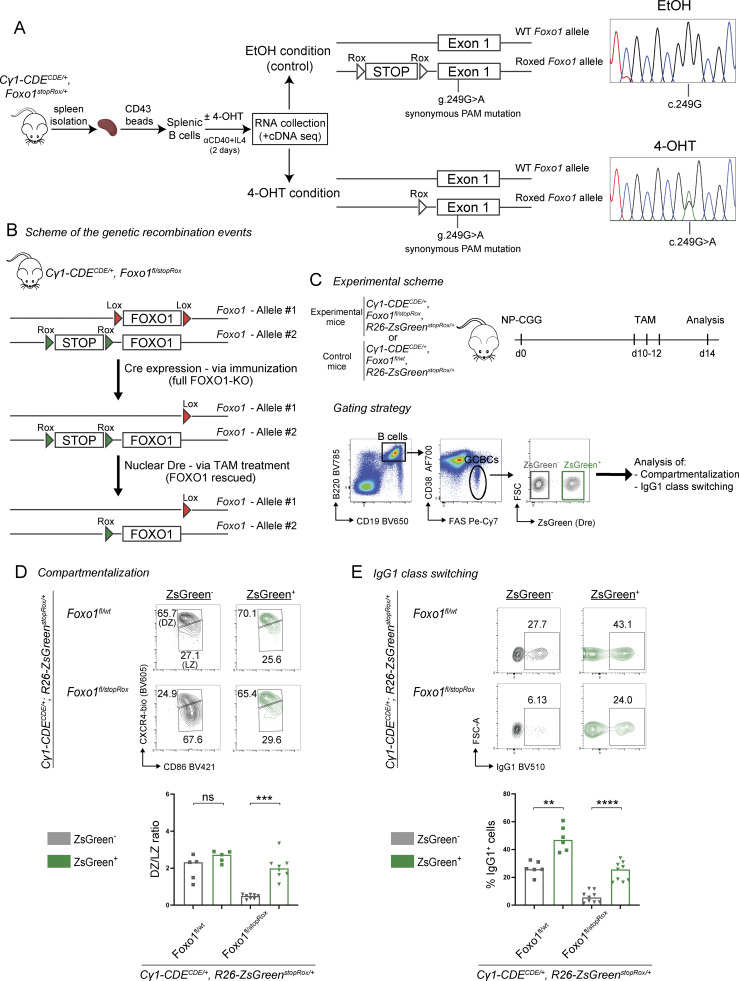
**Sequential mutagenesis at the endogenous *Foxo1* locus. (A)** Schematic representation of *in vitro* induction of the *Foxo1*^*stopRox*^ allele by *Cγ1-CDE* (left) and cDNA sequencing in activated *Cγ1-CDE, Foxo1*^*stopRox*^ splenic B cells (right). Expression of the targeted allele is observed only in the presence of 4-OHT, as demonstrated by the detection of the synonymous protospacer adjacent motif (PAM) mutation introduced during the targeting strategy of the transgenic strain. **(B)** Genetic scheme of recombination events after Cre and Dre activation at the *Foxo1* locus of *Foxo1*^*fl/stopRox*^ animals. **(C)** Experimental scheme (top) and representative gating strategy (bottom) employed for the phenotypic analysis of the GC compartment. **(D and E)** Top: representative flow cytometry plots measuring the percentage of DZ and LZ (D) and IgG1^+^ (E) splenic GCBCs of mice of the indicated genotypes at day (d)14 after immunization. Bottom: quantification of the DZ/LZ ratio (D) and the percentage of IgG1^+^ (E) ZsGreen^−^ (gray) and ZsGreen^+^ (green) splenic GCBCs. Statistics: Mann–Whitney test, **P ≤ 0.01; ***P ≤ 0.001; ****P ≤ 0.0001; ns, not significant. Each symbol represents one mouse. Data are from three independent experiments. Horizontal lines indicate the median.

The FOXO1 transcription factor, although dispensable for GC development/maintenance, positively regulates both GC compartmentalization (DZ compartment) and CSR (Dominguez-Sola et al., 2015; Sander et al., 2015). In view of this complex role in GCBC physiology, we aimed at genetically manipulating the endogenous *Foxo1* locus in a sequential manner, as a proof-of-concept experiment for our dual-recombinase strategy. In addition to the already published Cre-inducible *Foxo1* null allele (*Foxo1*^*fl*^) (Paik et al., 2007), we generated a rox-controlled, Dre-inducible *Foxo1* allele (*Foxo1*^*stopRox*^). The *Foxo1*^*stopRox*^ allele carries a STOP cassette flanked by rox sites in front of the Kozak sequence of *Foxo1* (labeled by a silent mutation in exon 1), leading to the inactivation of the allele in the absence of Dre activity (Fig. S2 A). Of note, while hemizygous *Foxo1* mice are viable and fertile, homozygous germline deletion of *Foxo1* results in embryonic lethality (Furuyama et al., 2004; Hosaka et al., 2004). In order to verify that the *Foxo1*^*stopRox*^ allele is functional upon deletion of the STOP cassette, splenic B cells from *Cγ1-CDE*, *Foxo1*^*stopRox/wt*^ compound mutant mice were activated *in vitro* and cultured in the presence or absence of 4-hydroxytamoxifen (4-OHT), followed by sequencing analysis of *Foxo1* transcripts (Fig. 2 A). Indeed, the expression of the targeted allele was observed solely in the presence of 4-OHT, demonstrating not only that the *Foxo1*^*stopRox*^ allele is functional, but also the tightness of the system. *In vivo*, crossing of *Cγ1-CDE*, *Foxo1*^*stopRox*^ mice with *Foxo1*^*fl*^ transgenic mice (*Cγ1-CDE*, *Foxo1*^*fl/stopRox*^) would generate a scenario in which upon NP-CGG immunization, and therefore Cre activation, the *Foxo1*^*fl*^ allele would be deleted, resulting in a Foxo1-KO condition in GCBCs (Fig. 2 B). GCBC-specific *Foxo1* deletion is expected to lead to loss of the DZ compartment and to defects in CSR, while maintaining the GCBC phenotype (Dominguez-Sola et al., 2015; Sander et al., 2015). In a second step upon TAM treatment—and hence Dre nuclear translocation and activation—the re-expression of FOXO1 would be achieved from the *Foxo1*^*stopRox*^ allele, thus leading to a rescue of the previously induced FOXO1 deficiency (Fig. 2 B). To address whether the sequential deletion and re-activation of *Foxo1* from the endogenous locus could restore GC compartmentalization and CSR, experimental *Cγ1-CDE*, *Foxo1*^*fl/stopRox*^*, R26-ZsGreen*^*stopRox*^ (in short *Foxo1*^*fl/stopRox*^) and control *Cγ1-CDE*, *Foxo1*^*fl/wt*^*, R26-ZsGreen*^*stopRox*^ (in short *Foxo1*^*fl/wt*^) mice were immunized with NP-CGG, subsequently treated with TAM (days 10-12 after immunization), and analyzed on day 14. The *R26-ZsGreen*^*stopRox*^ allele was included as an indirect reporter to identify Dre-recombined cells, i.e., FOXO1 re-expression in *Foxo1*^*stopRox*^ mice (Fig. 2 C). Strikingly, TAM administration with subsequent FOXO1 re-activation in the *Foxo1*^*fl/stopRox*^ group completely restored the DZ compartment in ZsGreen^+^ GCBCs, up to the same levels as in *Foxo1*^*fl/wt*^ control mice (Fig. 2 D and Fig. S2 B). This observation also suggests a high efficiency of Dre-mediated recombination in this setting, since all rox sites available (here the rox sequences in *Foxo1*^*stopRox*^ and *R26-ZsGreen*^*stopRox*^ alleles) are targeted in the same cell. In contrast, ZsGreen^−^ GCBCs in the experimental *Foxo1*^*fl/stopRox*^ group, of which the vast majority had failed to recombine the *Foxo1*^*stopRox*^ allele by Dre and had remained a full FOXO1-KO, lost the physiological levels of the DZ/LZ ratio, at the expense of DZ cells (Fig. 2 D) and in agreement with previous studies (Dominguez-Sola et al., 2015; Sander et al., 2015). Of note, the fraction of ZsGreen^+^ GCBCs in *Foxo1*^*fl/stopRox*^ mice was lower compared with that in controls, possibly reflecting the induction of FOXO1-associated apoptotic effects due to the sudden re-expression of FOXO1 (Dansen and Burgering, 2008; Srinivasan et al., 2009) (Fig. S2 C). Additionally, CSR to IgG1 was robustly restored in ZsGreen^+^ GCBCs in the *Foxo1*^*fl/stopRox*^ group, albeit at a lower frequency compared with controls (Fig. 2 E). We attribute this lower frequency to the fact that reporter ZsGreen^+^ cells from the *Foxo1*^*fl/stopRox*^ mice have expressed FOXO1 only for a maximum of 4 days (after TAM administration), in contrast to the control cells, in which FOXO1 is expressed from the beginning of the GC reaction. The higher frequency of CSR to IgG1 in control ZsGreen^+^ compared with control ZsGreen^−^ cells is due to the design of our Cre/DreERT2 line, since both IgG1 CSR and Dre-mediated ZsGreen recombination are dependent on activation of the *Cγ1* locus. Of note, not all control ZsGreen^+^ cells switched to IgG1 (Fig. 2 E), demonstrating that γ1 GLTs can be expressed independently of CSR (Casola et al., 2006; Roco et al., 2019).

### FOXO1-deficient GCBCs preserve their ability to class-switch upon FOXO1 re-expression

Under physiological conditions, CSR mostly occurs in pre-GCBCs, preceding SHM (Roco et al., 2019). Does the rescue of CSR observed following re-expression of FOXO1 at the peak of the GC reaction (Fig. 2, C and E) mainly occur in newly recruited GCBCs that populate the GC as late B cell incomers, rather than matured GCBCs? To address this issue, we performed a SHM analysis of the JH4 intronic region in GCBCs at day 14 after immunization. If CSR rescue predominantly happens in early GCBCs, the presence of mutations in *Foxo1*^*fl/stopRox*^ ZsGreen^+^ rescued cells should be scarce and lower than in control FOXO1-proficient (*Foxo1*^*fl/wt*^) and FOXO1-KO (*Foxo1*^*fl/stopRox*^ mice, which had not received TAM) cells. As expected, our SHM data showed a similar frequency of mutations between FOXO1-proficient and FOXO1-KO controls (Fig. S2 D), with only a slight increase in the fraction of highly mutated cells (<2% with ≥10 mutations) in the FOXO1-expressing control group (Sander et al., 2015). Similarly, the mutational load in ZsGreen^+^ (rescued) *Foxo1*^*fl/stopRox*^ cells was comparable to that in FOXO1-KO control cells (Fig. S2 D), suggesting that the rescue of CSR upon FOXO1 re-expression is predominantly reflecting CSR in already established and not newly recruited GCBCs.

In a second experimental approach, we asked whether CSR defects could also be rescued *in vitro* following the sequential KO and re-expression of FOXO1. To this end, we cocultured naïve B cells from control and experimental mice with 40LB feeder cells, a cell line that expresses CD40L and BAFF to mimic T cell–dependent immune responses and can robustly induce GC-like B cells (iGCBCs) in the presence of IL-4 (Nojima et al., 2011). Coculture with 40LB cells efficiently activates the *Cγ1* locus in B cells, inducing the Cre-mediated Foxo1-KO from the beginning of the experiment. Dre-mediated FOXO1 re-expression was stimulated by adding 4-OHT either at day 0—early FOXO1 induction upon B cell activation—or day 4—late FOXO1 induction in iGCBCs—of coculture, and CSR events were analyzed at day 4 or 8, respectively (Fig. S2 E). As expected, upon early rescue of FOXO1 at day 0, both FOXO1 levels and the frequency of class-switched iGCBCs were equivalent between control and rescued cells (Fig. S2, H and J) at day 4 of analysis. (FOXO1 was also detected in ZsGreen-negative cells, reducing the fidelity of the reporter as a readout in the *in vitro* setting.) Of note, control cells with one (heterozygous) or two (homozygous) copies of *Foxo1* were comparable in terms of class switching (Fig. S2, F and G). Strikingly, full rescue of CSR events was also detected at the late time point, i.e., when re-expression of FOXO1 was induced in cells already presenting a GC-like phenotype (Fig. S2 I). Together, these findings suggest that FOXO1-deficient GCBCs preserve the ability to undergo class switching if FOXO1 expression is enforced, both *in vitro* and *in vivo*, irrespective of the predominance of CSR at the pre-GCBC stage under physiological conditions (Roco et al., 2019).

### Re-induction of FOXO1 at the peak of the GC reaction restores the FOXO1-associated transcriptional program

**Figure 3.**
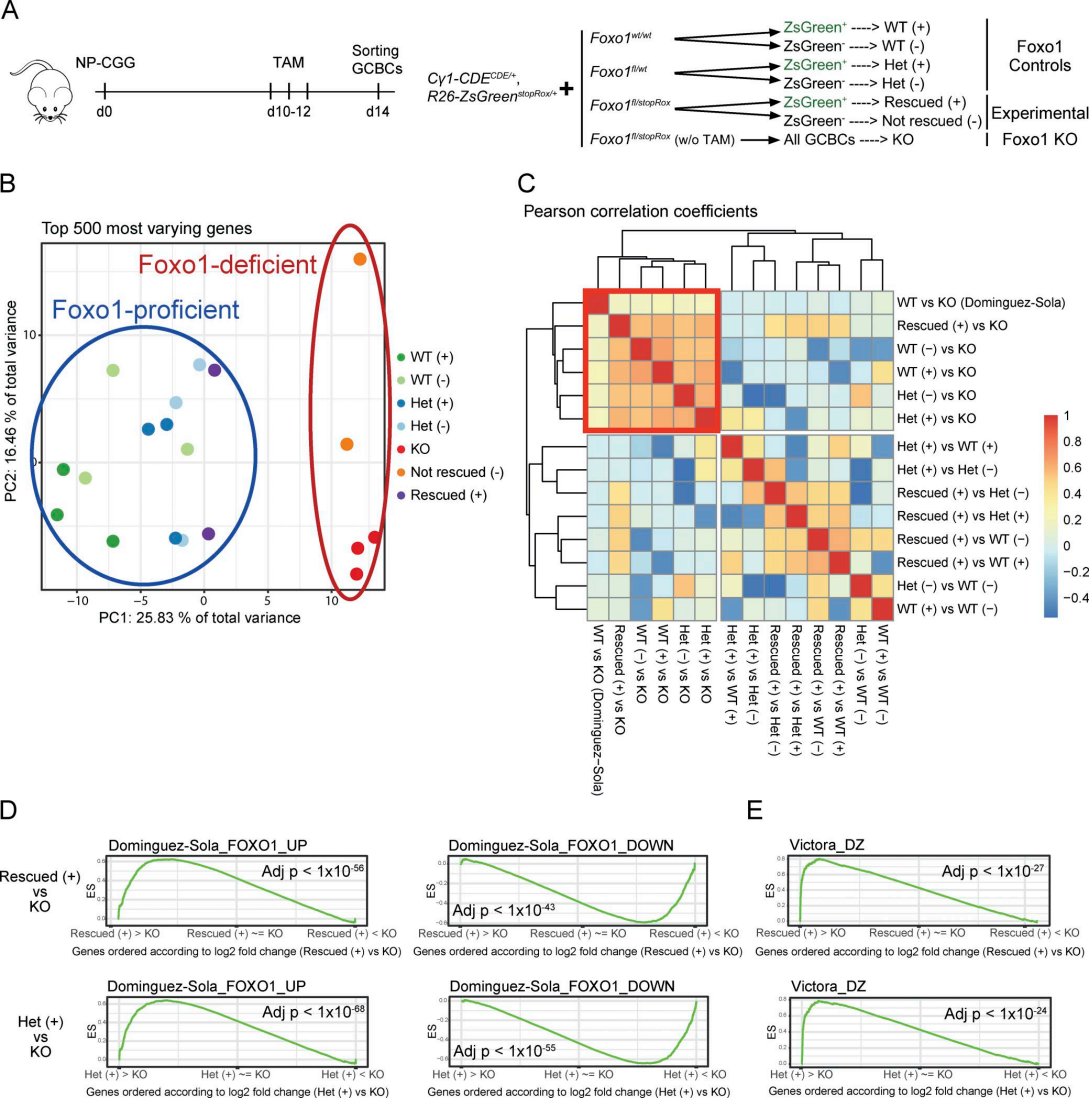
**Re-expression of FOXO1 at the peak of the GC reaction fully restores the FOXO1-associated transcriptional program in ZsGreen** ^**+**^ **rescued GCBCs. (A–E)** ZsGreen^+^ and ZsGreen^−^ GCBCs from experimental and control mice were sorted at day (d)14 after immunization (TAM d10–12) to perform RNA sequencing. **(A)** Experimental scheme (left) and overview of mouse genotypes (right, *n* = 2–3 mice per genotype). **(B)** PCA from the 500 most varying genes. Each dot represents one sample. **(C)** Heatmap showing Pearson’s correlation coefficients between log_2_ fold changes in gene expression profiles. **(D and E)** GSEA using published up- and downregulated Foxo1 (D) and DZ (E) gene signatures (Victora et al., 2012; Dominguez-Sola et al., 2015). Statistics: CERNO test. ES, enrichment score.

**Figure S3.**
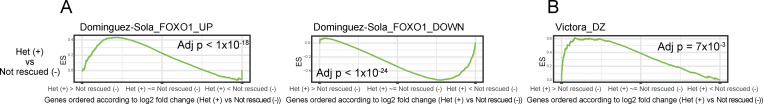
**Transcriptional profile of ZsGreen** ^**−**^ **(not-rescued) GCBCs resembles the FOXO1-KO transcriptional program. (A and B)** GSEA using published up- and downregulated Foxo1 (A) and DZ (B) gene signatures (Victora et al., 2012; Dominguez-Sola et al., 2015). Statistics: CERNO test. *n* = 2–3 mice per genotype. ES, enrichment score.

In order to investigate whether the re-expression of FOXO1 fully restores the FOXO1-dependent transcriptional program, reporter-positive and reporter-negative GCBCs of control *Foxo1*^*wt/wt*^ (*Foxo1* WT), *Foxo1*^*fl/wt*^ (*Foxo1* het), and experimental *Foxo1*^*fl/stopRox*^ mice were sorted at day 14 after immunization (TAM day 10–12) and subjected to RNA sequencing (Fig. 3 A; mRNA data available in NCBI Sequence Read Archive [SRA] database #PRJNA1365124). In addition, total GCBCs from *Foxo1*^*fl/stopRox*^ mice that had not received TAM were included, representing the FOXO1-KO situation. While the presence or absence of the ZsGreen reporter in control Foxo1-proficient (WT or het) GCBCs should show no differences, the expression status of the ZsGreen reporter in *Foxo1*^*fl/stopRox*^ experimental mice can be used to distinguish Foxo1-rescued (ZsGreen^+^) from cells, which failed to re-express FOXO1 (ZsGreen^−^, Foxo1 not-rescued). Principal component analysis (PCA) based on the top 500 genes showing the highest variability in expression across all samples clustered all Foxo1 control (WT and het) samples together—with no differences between reporter-positive and reporter-negative cells—while Foxo1-KO cells grouped separately (Fig. 3 B), indicating that the *Foxo1* expression level was the main determinant for differences between the samples (PC1) (Table S1). Importantly, ZsGreen^−^ cells from *Foxo1*^*fl/stopRox*^ experimental mice (no Foxo1 re-expression) clustered together with the Foxo1-KO controls, whereas the ZsGreen^+^ counterparts (Foxo1 re-expression by Dre^ERT2^-mediated recombination) grouped with the Foxo1 WT and het controls (Fig. 3 B), demonstrating that Foxo1-rescued cells present a similar transcriptional program compared with Foxo1 control cells. Similarly, a heatmap of Pearson’s correlation coefficients between log_2_ fold changes in gene expression profile showed positive correlations for multiple comparisons between Foxo1-KO vs. control Foxo1-proficient (WT or het) cells, including the independent dataset from Dominguez-Sola et al. (2015), comparing Foxo1 WT and KO cells (Fig. 3 C), corroborating a high degree of similarity between Foxo1 WT and Foxo1 het controls. Remarkably, the comparison between Foxo1-KO and ZsGreen^+^ rescued cells also correlated positively with the multiple comparisons between Foxo1-KO vs. Foxo1-proficient cells (Fig. 3 C), further indicating a similar transcriptome between rescued and WT/het control cells. In order to confirm that FOXO1 re-expression in ZsGreen^+^*Foxo1*^*fl/stopRox*^ cells results in restoration of the FOXO1-associated transcriptional profile, we performed gene set enrichment analysis (GSEA) (Subramanian et al., 2005) using Foxo1 transcriptional signatures (Dominguez-Sola et al., 2015) (Table S2). Since no differences were observed between all Foxo1-proficient control samples (Fig. 3, B and C), the ZsGreen^+^ Foxo1 het was chosen as Foxo1 control condition for the GSEA. Strikingly, Foxo1-rescued cells showed a highly significant enrichment of FOXO1-upregulated genes compared with Foxo1-KO cells, at levels comparable to the enrichment observed for Foxo1 control cells (Fig. 3 D, left panels), while FOXO1-downregulated genes were highly enriched in the Foxo1-KO cells (Fig. 3 D, right panels). Additionally, the DZ signature (Victora et al., 2012)—which is closely associated with Foxo1—was also significantly enriched among genes differentially expressed in rescued cells compared with Foxo1-KO cells, similar to the extent observed in control cells (Fig. 3 E, Table S2). Conversely, ZsGreen^−^ (not-rescued) cells displayed a pattern similar to the Foxo1-KO cells (Fig. S3, A and B). Taken together, the re-induction of FOXO1 at the peak of the GC reaction not only rescued the cells phenotypically, but also fully restored the transcriptional program associated with FOXO1, back to the levels of control cells.

## Discussion

We present proof-of-concept experiments that demonstrate the efficiency of sequential targeted mutagenesis through a novel dual-recombinase allele. This allele mediates cell type–specific expression of Cre recombinase, together with a TAM-regulated form of Dre recombinase (DreERT2) that can be subsequently activated in the same cells in a time-controlled manner. If successful, this approach allows for simplified breeding schemes for compound mutant mice, and promises high fidelity of sequential mutagenesis at the cellular level, in that Dre-mediated recombination is targeted and limited to the very cells that have previously been exposed to Cre activity.

In the *Cγ1-CDE* strain which we have constructed, Cre and DreERT2 are expressed from the 3′ UTR of the *Cγ1* locus that encodes the constant region of γ1 immunoglobulin heavy chain (Fig. 1 A). *Cγ1* GLTs are detected in the majority of B cells at the initiation of the GC reaction upon immunization with hapten-carrier conjugates, regardless of whether subsequent CSR to IgG1 takes place (Casola et al., 2006; Roco et al., 2019). Hence, highly efficient Cre- followed by Dre-mediated recombination was observed at the three time points analyzed, with the highest dual-labeling efficiency achieved at the earliest time point (day 7 after immunization), with up to 96% GCBCs expressing the Cre-dependent BFP reporter and more than half co-expressing Dre-dependent ZsGreen (Fig. 1 B). The lower frequency of Dre- as compared to Cre-mediated recombination may be due to the limited time window of TAM application. The reason for the lower labeling efficiencies at the later time points remains to be explored, but may simply reflect higher levels of *Cγ1* GLTs in GCBCs early after their recruitment. Dual labeling was also observed in a fraction of post-GC cells, such as MBCs and PCs (Fig. 1). In these compartments, the fractions of labeled cells varied depending on location and time of labeling after immunization, and while all BFP^+^ cells had presumably been labeled at the initiation of the GC response, ZsGreen labeling of some or most of these cells may have occurred already at the GC stage of differentiation. Unlabeled MBCs and PCs in these mice have likely arisen through pathways not involving an IL-4–rich environment and *Cγ1* transcription.

We used the *Cγ1-CDE* strain to analyze the role of the FOXO1 transcription factor at different stages of the GC reaction. In the absence of FOXO1, GCs exhibit a reduction of their fraction of proliferating cells, the so-called DZ, and lose their ability of undergoing CSR (Dominguez-Sola et al., 2015; Sander et al., 2015). Recent data suggest that the latter process is largely restricted to the initiation of the GC response (Roco et al., 2019). Accordingly, knocking out FOXO1 in *Cγ1-CDE* mice at the initiation of the GC response, through Cre-mediated recombination, resulted in GCs with a reduced DZ and drastically reduced CSR in GCBCs (Fig. 2). These phenotypes were fully rescued by Dre-mediated FOXO1 re-expression, with gene expression and SHM data indicating that this rescue occurred in matured GCBCs (Figs. 2, 3, and S2). These data indicate that GCBCs retain their ability to undergo CSR and respond to the corresponding signals, which apparently remain available at later stages of the GC reaction.

In summary, the *Cγ1-CDE* allele allows for highly efficient Cre- and subsequent Dre-mediated recombination in GCBCs. This tool can be used *in vivo* to study the interplay of distinct genetic events or the function of a specific gene at different time points during the GC reaction, and also to model the sequential acquisition of genetic alterations acquired during B cell lymphomagenesis, that often originates from GCBCs (Küppers et al., 1999). Similar dual-recombinase alleles could be profitably constructed for targeted sequential mutagenesis in other cellular contexts.

## Materials and methods

### Mice, immunization, and TAM treatment

*Cγ1-cre, R26-BFP* ^*stopF*^, and *Foxo1*^*fl*^ alleles have been described previously (Casola et al., 2006; Paik et al., 2007; Sommermann et al., 2020). *R26-ZsGreen*^*stopRox*^ is a derivative of the *R26-CAGS-lox-STOP-lox-rox-STOP-rox-ZsGreen* mice crossed to a *Deleter-Cre* line (Biglari et al., 2021). *Cγ1-CDE* and *Foxo1*^*stopRox*^ strains were generated by CRISPR/Cas9-mediated homologous recombination in C57BL/6 mouse zygotes according to previously published protocols (Weber et al., 2019). *Cγ1-CDE* and *Foxo1*^*stopRox*^ strains are available at the Jackson Laboratory Repository with the JAX Stock No. 040961 and 040962, respectively.

Mice were bred and maintained under specific pathogen–free conditions. 8–15-week-old male or female mice were immunized with 100 µg alum-precipitated NP-CGG (ratio 10-19; Cat#N-5055B; LGC Biosearch Technologies) intraperitoneally followed by TAM administration at the indicated time points (4 mg/day oral gavage, TAM; #T5648-5G, 99%; Sigma-Aldrich). Experimental animal procedures were approved by the Landesamt für Gesundheit und Soziales Berlin (G0308/19, G0062/21).

### Flow cytometry

Cells from spleen and bone marrow were collected in B cell medium (DMEM supplemented with 10% FCS, 1× non-essential amino acids (NEAA), 1 mM sodium pyruvate, 2 mM L-glutamine, 1 mM HEPES, 1× penicillin–streptomycin, 50 µM β-ME). Erythrocytes were lysed with Gey’s solution, and single-cell suspensions were stained with antibody conjugates in PBS, pH 7.2, supplemented with 0.5% BSA and 2 mM EDTA. For intracellular staining, single-cell suspensions were stained with Foxp3/Transcription Factor Staining Buffer Set (#00-5523-00; eBioscience), according to the manufacturer’s instructions. Zombie Aqua Fixable Viability Kit (#423102; BioLegend) was used to assess live/dead status. The following antibodies were used: B220-BV785 (clone RA3-6B2; #103246; RRID #AB_2563256; BioLegend), CD19-BV650 (clone 6D5; #115541; RRID #AB_11204087; BioLegend), CD38-AF700 (clone 90; #56-0381-82; RRID #AB_657740; eBioscience), CD86-BV421 (clone GL-1; #105032; RRID #AB_2650895; BioLegend), CD86-PE-Cy7 (clone GL-1; #105014; RRID #439783; BioLegend), CD95/FAS-PE (clone Jo2; #554258; RRID #AB_395330; BD Biosciences), CD95/FAS-PE-Cy7 (clone Jo2; #557653; RRID #AB_396768; BD Biosciences), CXCR4-bio (clone 2B11; #13-9991-82; RRID #AB_10609202; Invitrogen), FOXO1-AF647 (clone C29H4; #72874; RRID #AB_2799829; Cell Signaling), GL7-PE (clone GL7; #144608; RRID #AB_2562926; BioLegend), IgE-BV605 (clone R35-72; #744281; RRID #AB_2742118; BD Biosciences), IgG1-APC (clone A85-1; #560089, RRID #AB_1645625; BD Biosciences), IgG1-BV510 (clone A85-1; #740121; RRID #AB_2739879; BD Biosciences), IgG1-PE (clone A85-1; #550083; RRID #AB_393553; BD Biosciences), IgM-bio (clone Il/41; #13-5790-81; RRID #AB_466675; eBioscience), and Streptavidin-BV605 (#405229; BioLegend). Samples were analyzed on an LSRFortessa (BD Biosciences). Plots were generated using FlowJo software (BD FlowJo) and graphs by Prism software (GraphPad Prism).

### cDNA sequencing of targeted *Foxo1*^*stopRox*^ allele

Transgenic splenic B cells were isolated by CD43 depletion with magnetic anti-mouse CD43 microbeads (Cat#130-049-081; Miltenyi Biotec) according to the manufacturer’s instructions. To remove the rox-flanked STOP cassette, B cells were activated with IL-4 (25 ng/ml; Cat#404-ML; R&D) and αCD40 (1–2 µg/ml; HM40-3; Cat#102908; BioLegend) and cultured in the presence of 4-OHT (1 µM; Cat#H6278; Sigma-Aldrich). 3 days later, total RNA was extracted using AllPrep DNA/RNA Mini Kit (#80204; Qiagen) according to the manufacturer’s instructions. For cDNA synthesis, 500 ng of RNA was used per reaction and reverse transcription was performed with SuperScript II Reverse Transcriptase (#18064014; Invitrogen). A mixture of random and oligo(dT) primers was used following the manufacturer’s protocol. PCR amplification of *Foxo1* cDNA was performed using the following primers: forward 5′-ATG​GCC​GAA​GCG​CCC​CAG​GTG​GTG​GAG​AC-3′ and reverse 5′-CCT​ACT​TCA​AGG​ATA​AGG​GCG​ACA​GCA​AC-3′. PCR products were gel-purified using the NucleoSpin Gel and PCR Clean-Up kit (#740609.250; Macherey-Nagel) and sequenced by Sanger sequencing.

### 40LB *in vitro* coculture

40LB feeder cells (Nojima et al., 2011) were maintained in DMEM supplemented with 10% FCS, 1× penicillin–streptomycin, 1 mM sodium pyruvate, and 1× GlutaMAX. 40LB cells were irradiated with 20 Gγ and cocultured with isolated splenic naïve B cells (supplemented with 1 ng/ml IL-4 and in the presence or absence of 1 µM 4-OHT) to induce GC-like B cells.

### SHM analysis

GCBCs of the indicated genotypes were sorted (FACSAria, BD Biosciences) and stored at −70°C. DNA was extracted using the AllPrep DNA/RNA micro kit (#80284; Qiagen), and JH4 intronic region was PCR-amplified using the KOD polymerase (#71086-3; Sigma-Aldrich) using the following primers: VHA/VH1 forward primer 5′-ARGCCTGGGRCTTCAGTGAAG-3′, VHE/VH5 forward primer 5′-GTG​GAG​TCT​GGG​GGA​GGC​TTA-3′, and JH4 intronic reverse primer 5′-CTC​CAC​CAG​ACC​TCT​CTA​GAC​AGC-3′. JH4 PCR products (800 bp) were purified by gel electrophoresis and extracted using the NucleoSpin Gel and PCR Clean-Up kit (#740609.250; Macherey-Nagel). Purified products were cloned into the pCR4Blunt-TOPO vector according to manufacturer’s instructions (Zero Blunt TOPO PCR Cloning Kit, #450031; Invitrogen), and transformation was carried out using Top10 competent cells. 30-40 colonies were cultured overnight, and DNA plasmids were isolated (NucleoSpin Plasmid DNA Purification Kit; #740588.250; Macherey-Nagel) and sent for Sanger sequencing (T7 primer, LGC Genomics). Sequences were aligned using Basic Local Alignment Search Tool to quantify the number of somatic mutations.

### RNA sequencing

5,000 GCBCs of the indicated genotypes were sorted (FACSAria, BD Biosciences) into 75 μl RLT plus (RNeasy lysis) buffer (with β-mercaptoethanol) and stored at −70°C. RNA was extracted using the AllPrep DNA/RNA micro kit (#80284; Qiagen), and a low-input protocol for library preparation was conducted as previously described (Rosales et al., 2018). In short, cDNA was synthesized using SuperScript II Reverse Transcriptase (#18064022; Invitrogen), together with Template Switching Oligo and Oligo(dT)-30VN, followed by full-length cDNA amplification using KAPA HiFi HotStart Ready Mix (#KK2601; Roche) and an IS-PCR primer. cDNA libraries were prepared using the Nextera XT DNA library preparation kit (#FC-131-1024; Illumina) and Illumina DNA/RNA UD Indexes Set A with Tagmentation kit (#20091654; Illumina), following manufacturer’s instructions. Libraries were sequenced on a NovaSeq X Plus 25B instrument as paired-end 150-bp reads with a depth of 100 million reads per sample.

Reads were trimmed using bbduk v39.08 for adapters, poly-G tails, and 5′ end nucleotide composition bias due to shearing. RNA quantification was performed with Salmon v1.10.3 using GRCm39 as a mouse reference genome and using GENCODE version M36 for basic feature annotations. Gene-based expression levels were computed using the Bioconductor package tximport v1.34, and the normalization and differential expression using the Bioconductor package DESeq2 v1.46, on all genes with at least 5 reads in at least 3 samples. The log2 fold changes were moderated using the R package ashr v2.2.63. The expression values used for the PCA plot were normalized with the variance-stabilized transformation implemented in DESeq2. The transcriptional profile of one *Foxo1*^*fl/stopRox*^ mouse revealed an underlying infection unrelated to the experiment, which precluded its inclusion in the final analysis. The gene set enrichment running scores were computed with the Bioconductor package DOSE version 4.2.0, and their statistical significance was assessed using the CERNO test (Yamaguchi et al., 2008). All differential expression analysis, tables, and figures were generated using R version 4.4.3 (differential expression) and 4.5.0 (tables and figures).

### Online supplemental material

Fig. S1 shows additional characterization of the *Cg1-CDE* strain. Fig. S2 shows additional characterization of the sequential mutagenesis at the *Foxo1* locus (Cre-mediated FOXO1 KO followed by Dre-mediated re-expression of FOXO1). Fig. S3 shows the GSEA using Foxo1 and DZ signatures comparing FOXO1-proficient vs. ZsGreen^−^ (not-rescued) *Foxo1*^*fl/stopRox*^ conditions. Table S1 shows differentially expressed genes between multiple comparisons. Table S2 shows GSEA results.

## Supplementary Material

Table S1 shows differentially expressed genes between multiple comparisons.

Table S2 shows GSEA results.

## Data Availability

The mRNA sequencing data are publicly available in the NCBI SRA database (accession number PRJNA1365124).
